# GSI CTA evaluation of the vertebrobasilar artery in normal adults at high altitude

**DOI:** 10.3389/fcvm.2023.1094401

**Published:** 2023-05-24

**Authors:** Jia Wei, Huiying Hu, Xin He, Haihua Bao

**Affiliations:** Department of Medical Imaging Center, Qinghai University Affiliated Hospital, Xining, China

**Keywords:** high altitude, vertebrobasilar morphology, geometry, energy-spectrum CT, posterior circulation

## Abstract

**Objective:**

Vascular geometry is influenced by several factors during its growth and development. Here, we compared the differences in vertebrobasilar geometry among residents of a plateau region at different altitudes and investigated the relationship between vascular geometry and altitude.

**Methods:**

Data of some adults in the plateau region who experienced vertigo and headache as the main symptoms but had no evident abnormalities found during imaging examination were collected. They were divided into three groups based on an altitude gradient: group A (1,800–2,500 masl), group B (2,500–3,500 masl), and group C (over 3,500 masl). They underwent head–neck energy-spectrum computed tomography angiography with a gemstone spectral imaging scanning protocol. The following indices were observed: (1) vertebrobasilar geometric configurations (walking, tuning fork, lambda, and no confluence), (2) vertebral artery (VA) hypoplasia, (3) the bending number of bilateral VA intracranial segment, (4) length and tortuosity of the basilar artery (BA), and (5) anteroposterior (AP)–mid–BA angle, BA–VA angle, lateral–mid–BA angle, and VA–VA angle.

**Results:**

Of the 222 subjects, 84 of them were included in group A, 76 in group B, and 62 in group C. The number of participants in walking, tuning fork, lambda, and no confluence geometries was 93, 71, 50, and 8, respectively. As altitude increased, the tortuosity of the BA also increased (1.05 ± 0.06 vs. 1.06 ± 0.08 vs. 1.10 ± 0.13, *P *= 0.005), as did the lateral–mid–BA angle (23.18° ± 9.53° vs. 26.05° ± 10.10° vs. 31.07° ± 15.12°, *P *= 0.007) and the BA–VA angle (32.98° ± 17.85° vs. 34.51° ± 17.96° vs. 41.51° ± 19.22°, *P *= 0.024). There was a relatively weak positive correlation between the altitude and the tortuosity of the BA (*r*_s _= 0.190, *P *= 0.005), the lateral–mid–BA angle (*r*_s _= 0.201, *P *= 0.003), and the BA–VA angle (*r*_s _= 0.183, *P *= 0.006) which showed a significant difference. Compared with groups A and B, there were more multibending groups and fewer oligo-bending groups in group C (*P *< 0.001). There was no difference found in the vertebral artery hypoplasia, actual length of the BA, VA–VA angle, and AP–mid–BA angle among the three groups.

**Conclusion:**

As the altitude increased, the tortuosity of the BA and the sagittal angle of the vertebrobasilar arterial system also increased. The increase in altitude can lead to changes in vertebrobasilar geometry.

## Introduction

Posterior circulation ischemia (PCI) accounts for approximately 20%–25% of ischemic strokes (1). Posterior circulation stroke (PCS) has higher mortality and morbidity compared with anterior circulation ischemic stroke (2), and large artery atherosclerosis is one of the most common stroke mechanisms of non-cardiogenic PCS (3). The morphology of the vertebrobasilar arterial system (VAS) plays an important role in the formation and development of atherosclerotic plaques and may even affect the blood supply of posterior circulation resulting in PCI (4). The morphology and course of the vertebrobasilar artery are highly diverse due to the influence of many factors during growth and development. A study indicated that the diameter, length, position, and congenital variation of the vertebrobasilar artery depend on several factors such as sex, age, and race (5). However, the effect of long-term plateau hypoxic environment on morphology has not been reported. The patients underwent energy-spectrum computed tomography (CT) angiography by using optimal single-energy imaging combined with individualized contrast injection protocols. This angiography can improve image quality while effectively reducing contrast dose and radiation dose (6). In this study, we used energy-spectrum CT angiography (CTA) to analyze the morphological structure of VAS in normal adults at different sea levels in the plateau region, to investigate the relationship between vascular geometry and plateau hypoxic environment, and to provide a certain reference basis for the understanding of cerebrovascular diseases at high altitude.

## Materials and methods

### Study population

A number of patients at high altitude who were admitted to a tertiary general hospital (Xining, Qinghai Province, China, with an elevation of approximately 2,250 masl) and underwent head–neck CTA between January 2020 and April 2022 were selected to be included in this retrospective study. The inclusion criteria were the following: (a) All participants were residents of Qinghai Province (with altitude range from 1,800 masl in Minhe County to 4,403 masl in Gado County), and these residents were regarded as those who lived in the Qinghai Province region for more than 5 years, although more than 80% of the them specified that they were born and had permanently lived in Qinghai Province. (b) The patients experienced suspected symptoms of PCI such as vertigo, headache, diplopia, blurred vision, and tinnitus, but had no evident abnormalities found during imaging examination. (c) From entering the plateau to receiving hospital treatment, all patients did not leave the high-altitude environment. The exclusion criteria were the following: (a) The patients had a history of cerebrovascular diseases, such as cerebral aneurysm, intracranial vascular stenosis, arteritis and Moyamoya disease, cerebral infarction, cerebral hemorrhage, etc. (b) The patients had chronic diseases affecting the normal blood supply of cerebral vessels, such as diabetes, hyperlipidemia, and hypertension. (c) They were allergic to the contrast medium and suffered from claustrophobia. (d) Pregnant women were not allowed in this study. (e) Poor image quality of the examination results, which cannot meet the diagnostic requirements, was also a factor.

### Patients group

Based on the altitude gradient, the patients were divided into three groups: group A (1,800–2,500 masl), group B (2,500–3,500 masl), and group C (≥3,500 masl). The morphological characteristics of the vertebrobasilar artery being measured are as follows.

### Image acquisition

All head–neck CTAs were performed using a 256-row spiral CT scanner (Revolution CT, GE Healthcare, Milwaukee, USA) with a gemstone spectral imaging (GSI) scanning protocol. The scan parameters were as follows: (a) tube voltage, rapid dual kVp (80 and 140 kVp) switching in 0.25 ms; (b) tube current, GSI Assist mA modulation (320 mA); and (c) 0.5 s tube rotation time with a pitch factor of 0.984:1 (25 cm DFOV). The coronal scanning coverage extended from the aortic arch to the cranial vault. The contrast agent (Ultravist-370, 370 mg/ml, Bayer Healthcare, Berlin, Germany) was injected into the right antecubital vein at 4.5 ml/s (median volume: 0.8 ml/kg), followed by a saline flush of 40 ml at 5.0 ml/s. SmartPrep tracking technology was used, and the region of monitoring was placed at the center of the descending aorta with a threshold value of 100 HU to trigger the scan with a 2.7 s scan delay. All images were reconstructed at 0.625 mm slice thickness by using the adaptive statistical iterative reconstruction (ASiR) algorithm to reduce the radiation dose requirement, the strength of ASiR was 60%, and the energy level of 40–60 keV was selected for reconstruction.

### Measurement of vascular geometry

**Figure 1:**
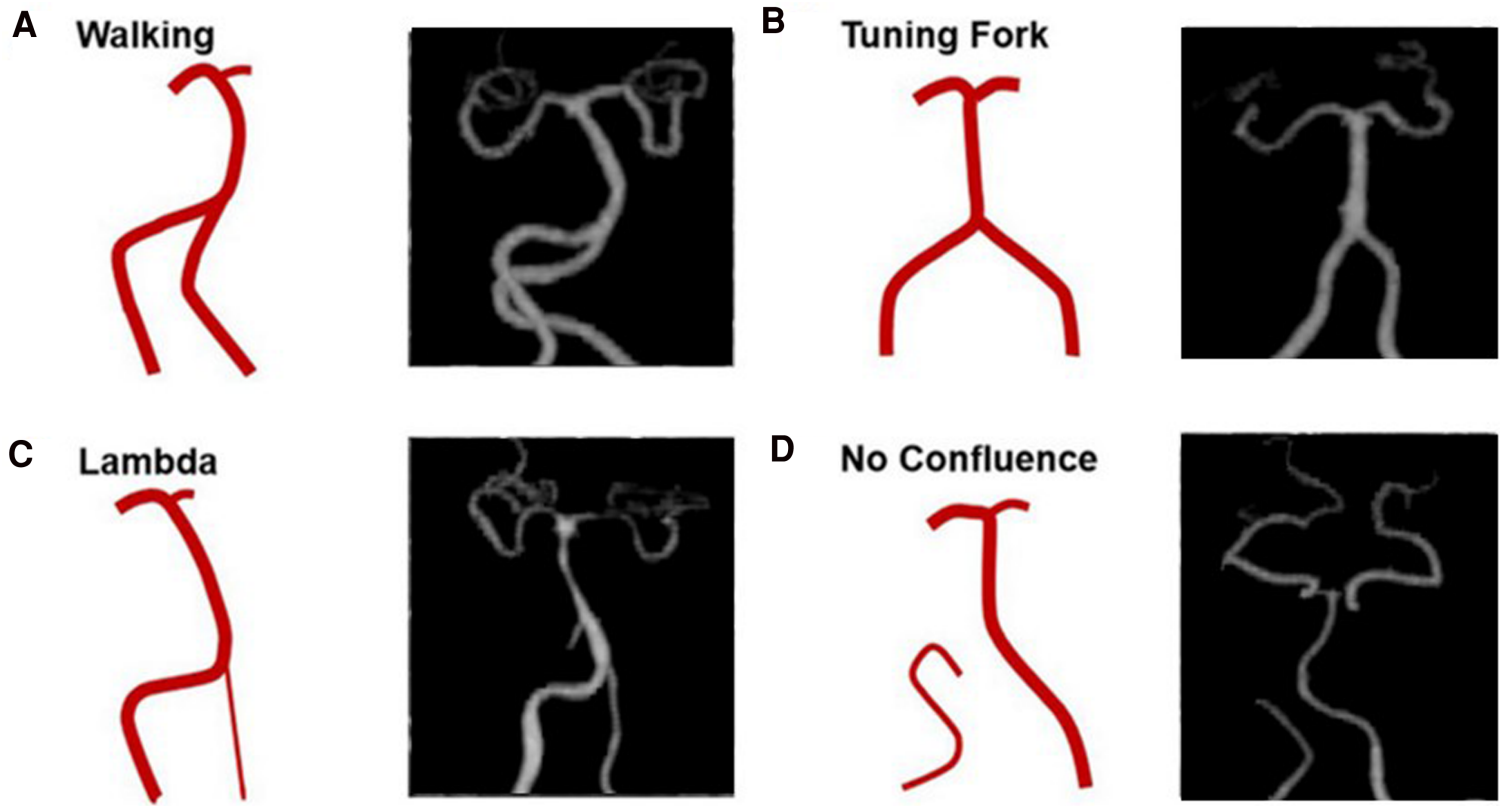
Four basic geometric configurations of vertebrobasilar artery: walking (**A**), tuning fork (**B**), lambda (**C**), and no confluence (**D**) (cited from Zheng et al., (8)).

**Figure 2:**
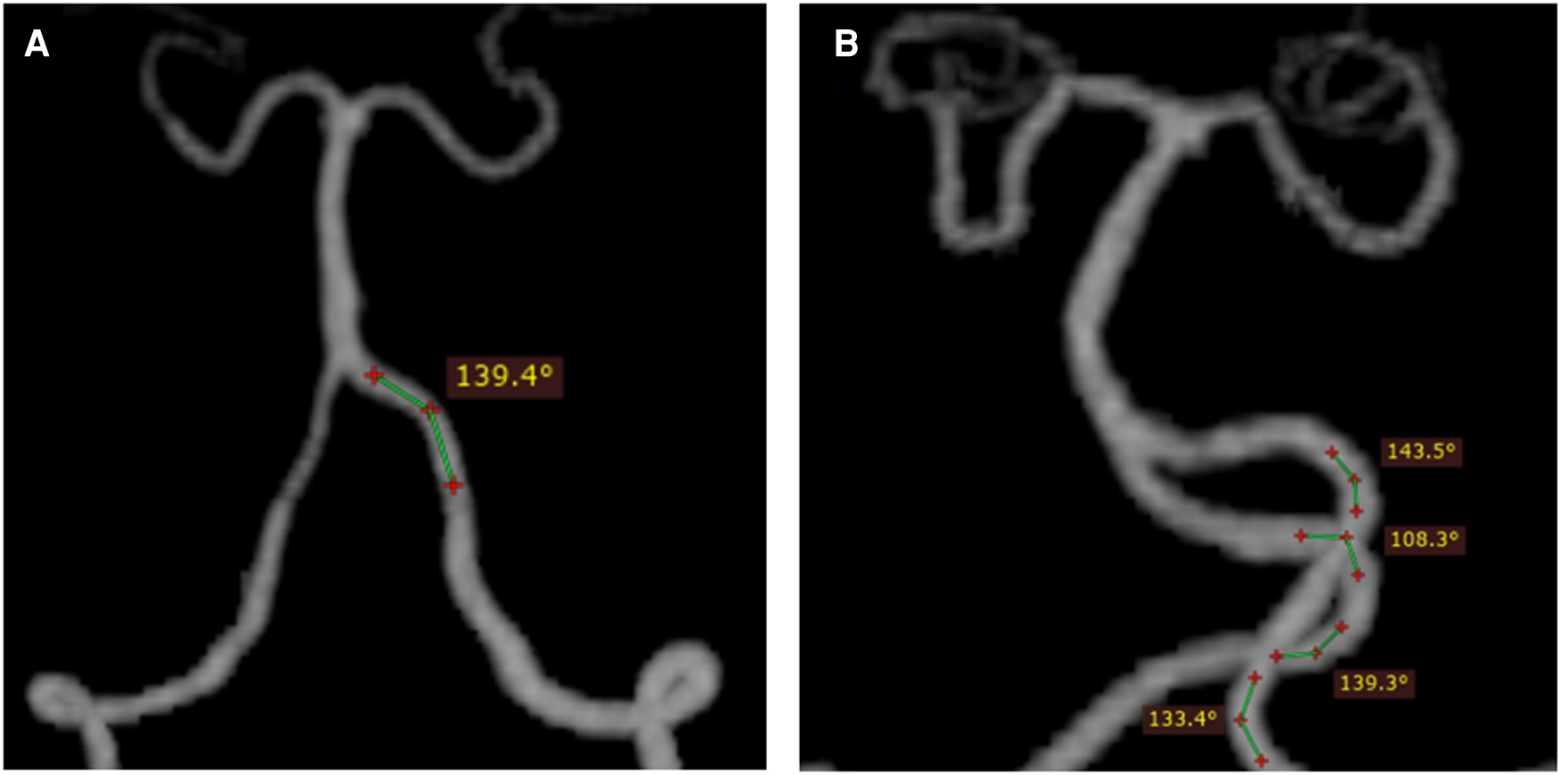
Schematic diagram of vascular curvature in the vertebral artery: oligo-bending group (the number of vascular bends <3) (**A**) and multibending group (the number of vascular bend ≥3) (**B**). From the vertex of a vascular curve, two lines were drawn to both sides. If the angle formed by the intersection of the two lines with the VA was ≤150°, a vascular bend was identified.

**Figure 3:**
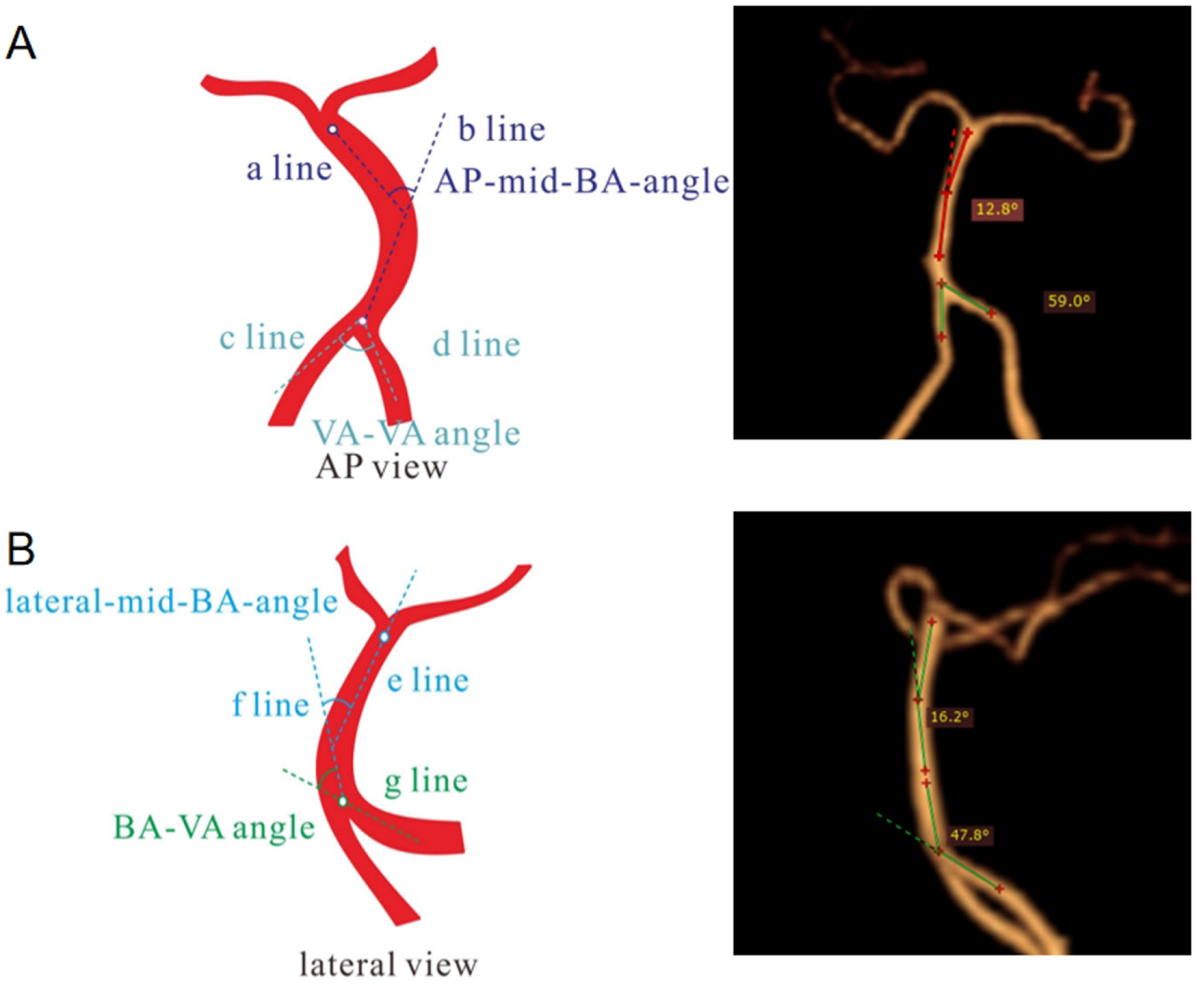
Illustration of the measurement of basilar artery geometry. In the AP view (**A**), the angle between VAs (a line and b line) was considered the AP–mid–BA angle, and the angle between c line and d line was considered the VA–VA angle. In the lateral view (**B**), the angle between e line and f line was considered the lateral-Mid–BA angle, and the angle between the BA (f line) and the dominant VA (g line) was regarded as the lateral BA–VA angle. AP, anteroposterior; BA, basilar artery; VA, vertebral artery.

The images were imported into a medical image processing software (Cerebral Go) and RadiAnt DICOM Viewer 5.0.1 software to observe and semiautomatically measure the morphological characteristics of the vertebrobasilar artery. The measurement indicators were as follows: (a) The basic geometric configurations of vertebrobasilar artery were walking (7), tuning fork (7), lambda (7), and no confluence (8), as shown in Figure 1. (b) If an angle of the curvature of vertebral artery (VA) was ≤150°, it was defined as a vascular bend; according to the total number of vascular bends in the bilateral VA intracranial segment, the patients were divided into a multibending group (the number of vascular bends ≥3) and an oligo-bending group (the number of vascular bends <3) (8), as presented in Figure 2. (c) Vertebral artery hypoplasia (VAH) was found to have a unilateral vertebral artery V4 segment diameter of ≤2.0 mm (9). (d) The actual length of the basilar artery (BA), the straight length of BA (10), the calculation of the tortuosity of BA (10) was found to be the actual length/the straight length^−1^ × 100%. (e) The direction of BA convex curvature was defined to be the right convex, left convex, and straight BA (11). (f) From the AP view, two lines were drawn from the midpoint of the BA to the apex of the BA (posterior cerebral artery side) and the union point of the VAs on both sides, and the angle between the two lines was defined as the AP–mid–BA angle (11). The angle measured by the same method on the lateral view is the lateral–mid–BA angle (11). From the lateral view, the angle between the BA and dominant VA is the BA–VA angle (11), as demonstrated in Figure 3. (g) The VA–VA angle is the angle between the two vertebral arteries. The vascular geometry was measured by two experienced radiologists, and any inconsistencies were discussed and then unified.

### Statistical analysis

Quantitative data of normal distribution were expressed as the mean ± standard deviation (SD), and the comparison among the three altitude groups was performed by using the ANOVA test. Quantitative data of non-normal distribution were expressed as median (interquartile range, IQR), and the comparison among the groups was carried out by using the Kruskal–Wallis test. Qualitative data were expressed as numbers (*n*) or percentages (%), and the comparison among the groups was conducted by using the Chi-square test. In addition, two-by-two comparisons were made in the data with a statistically significant difference. A Spearman correlation analysis was estimated to determine the linear relationship between the altitude and various morphological indexes of the vertebrobasilar artery. The *P*-value < 0.05 was considered statistically significant. All statistical analyses were performed using the SPSS software for Windows (version 22.0; SPSS Inc.).

## Results

### Basic information of groups at different altitudes

A total of 222 patients were included in this study, of whom 119 (53.6%) were male and 103 (46.4%) were female. Their mean age was 51.1 ± 14.8 years. Based on the altitude gradient, they were classified into three groups: 84 patients (37.8%) in group A (1,800–2,500 masl), 76 (34.2%) in group B (2,500–3,500 masl), and 62 (27.9%) in group C (≥3,500 masl). The average altitude of these three groups was 2,156.81 ± 169.15 masl; 2,896.11 ± 233.93 masl; and 3,856.53 ± 236.273 masl, respectively. There was significant difference in age in the baseline characteristics among the three groups (*P* < 0.001). The detailed information is presented in Table 1.

**Table 1:** Demographic characteristics of the study subjects in three altitude groups.

	Group A (1,800–2,500 m) (*N* = 84)	Group B (2,500–3,500 m) (*N* = 76)	Group C (≥3,500 m) (*N* = 62)	*P*-value
Sex				0.095
Male	49 (58.3%)	44 (57.9%)	26 (41.9%)	
Female	35 (41.7%)	32 (42.1%)	36 (58.1%)	
Age, years	55.0 (51.0, 64.0)	53.0 (42.5, 60.5)	43.0 (32.0, 54.0)	**0**.**000**
Current smoker	26 (31%)	30 (39.5%)	18 (29.0%)	0.364
Alcohol drinking	24 (28.6%)	26 (34.2%)	20 (32.3%)	0.738
Race				0.070
Han population	45 (53.6%)	35 (46.1%)	23 (37.1%)	
Hui population	19 (22.6%)	13 (17.1%)	10 (16.1%)	
Tibetan	20 (23.8%)	28 (36.8%)	29 (46.8%)	
Average altitude, masl	2,250.0 (2,012.5, 2,250.0)	2,880.0 (2,720.0, 2,980.0)	3,730.0 (3,710.0, 4,020.0)	**0**.**000**

Bold values denote statistical significance (*P* < 0.05).

### Distribution of basic geometric configurations of the vertebrobasilar artery

In this study, the walking, tuning fork, lambda, and no confluence geometries accounted for 41.9% (93 patients), 32.0% (71 patients), 22.5% (50 patients), and 3.6% (8 patients), respectively. The mean VA–VA angles for walking, tuning fork, and lambda were 56.27° ± 18.83°, 65.48° ± 26.31°, and 62.74° ± 24.85°, respectively, with no comparison differences found (Table 2).

**Table 2:** Distribution of basic geometric configurations of the vertebrobasilar artery.

	Walking (*N* = 93)	Tuning fork (*N* = 71)	Lambda (*N* = 50)	No confluence (*N* = 8)	*P*-value
Altitude					0.130
Group A	34 (36.6%)	29 (40.8%)	21 (42.0%)	0 (0.0%)	
Group B	26 (28.0%)	23 (32.4%)	20 (40.0%)	7 (87.5%)	
Group C	33 (35.4%)	19 (26.8%)	9 (18.0%)	1 (12.5%)	
VA–VA angle (°)	52.50 (43.70,70.45)	64.50 (44.50,74.60)	61.15 (41.73,74.53)	/	0.061

VA, vertebral artery.

### Comparison of BA and vertebrobasilar morphology in the three altitude groups

The mean actual length of the BA was 2.53 cm ± 0.44 cm, and the mean tortuosity of the BA was 1.07 ± 0.10. As the altitude increased, the tortuosity of the BA also increased (1.05 ± 0.06 vs. 1.06 ± 0.08 vs. 1.10 ± 0.13, *P *= 0.005). The lateral–mid–BA angle (23.18° ± 9.53° vs. 26.05° ± 10.10° vs. 31.07° ± 15.12°, *P *= 0.007) and the BA–VA angle (32.98° ± 17.85° vs. 34.51° ± 17.96° vs. 41.51° ± 19.22°, *P *= 0.024) significantly increased, and after a pairwise comparison, the differences of the lateral–mid–BA angle (*P *= 0.005) and the BA–VA angle (*P *= 0.023) at altitude were mainly observed between group A and group C. Compared with the tortuosity of groups A and B, the tortuosity of BA significantly increased in group C (*P *= 0.010; *P *= 0.016). However, there was no difference found in the direction of BA convex curvature, the actual length of BA, and the AP–mid–BA angle between the three groups (Table 3).

**Table 3:** Comparison of BA and vertebrobasilar morphology in the three altitude groups.

	Group A (1,800–2,500 m) (*N* = 84)	Group B (2,500–3,500 m) (*N* = 76)	Group C (≥3,500 m) (*N* = 62)	*P*-value
Direction of BA convex curvature				0.224
Left convex	26 (31.0%)	29 (38.2%)	27 (43.5%)	
Right convex	42 (50.0%)	32 (42.1%)	30 (48.4%)	
Straight	16 (19.0%)	15 (19.7%)	5 (8.1%)	
Actual length (cm)	2.41 (2.24, 2.71)	2.46 (2.23, 2.82)	2.51 (2.25, 2.74)	0.688
Straight length (cm)	2.37 ± 0.32	2.42 ± 0.35	2.31 ± 0.37	0.397
Tortuosity	1.03 (1.01, 1.07)	1.03 (1.01, 1.08)	1.06 (1.03, 1.13)^a,b^	**0**.**005**
AP–mid–BA angle (°)	22.80 (13.55, 32.65)	22.10 (10.10, 34.25)	27.70 (14.80, 43.35)	0.059
Lateral–mid–BA angle (°)	23.25 (16.53, 29.38)	23.60 (18.35, 32.45)	27.60 (20.70, 39.20)^a^	**0**.**007**
BA–VA angle (°)	31.35 (20.03, 45.13)	33.30 (21.70, 46.75)	34.80 (29.95, 53.50)^a^	**0**.**024**

BA, basilar artery; VA, vertebral artery; AP, anteroposterior.

Bold values denote statistical significance (*P* < 0.05). In the groups of BA tortuosity: group A vs. group B, *P' *= 1.0; group A vs. group C, *P' *= 0.010; and group B vs. group C, *P' *= 0.016. In the groups of lateral–mid–BA angle: group A vs. group B, *P' *= 0.318; group A vs. group C, *P' *= 0.005; and group B vs. group C, *P' *= 0.334. In the groups of BA–VA angle: group A vs. group B, *P' *= 1.0; group A vs. group C, *P' *= 0.023; and group B vs. group C, *P' *= 0.158. The significant level was adjusted to *P'*, *P'* = *p*/*m*, *m* = *k* (*k* − 1)/2 + 1, *k* indicates the group number, and *P'* ≤ 0.05 was considered to be statistically significant.

^a^ Groups B and C were compared with group A (*P'* ≤ 0.05).

^b^ Group B was compared with group C (*P* ≤ 0.05).

### Comparison of VA morphology in the three altitude groups

There were more multibending groups and fewer oligo-bending groups in group C (*P *< 0.001) compared with groups A and B, and there had been no difference observed between group A and group B, while there was also no difference in the comparisons of VAH and the VA–VA angle between the three groups (Table 4).

**Table 4:** Comparison of VA morphology in the three altitude groups.

	Group A (1,800–2,500 m) (*N* = 84)	Group B (2,500–3,500 m) (*N* = 76)	Group C (≥3,500 m) (*N* = 62)	*P*-value
VAH				0.233
Right	15 (17.9%)	17 (22.4%)	9 (14.5%)	
Left	11 (13.1%)	14 (18.4%)	5 (8.1%)	
Normal	58 (69.0%)	45 (59.2%)	48 (77.4%)	
Bending number of bilateral VA intracranial segment				**0**.**000**
Multi-bending	39 (46.4%)^a^	29 (38.2%)^a^	44 (71.0%)^b^	
Oligo-bending	45 (53.6%)^a^	46 (61.8%)^a^	18 (29.0%)^b^	
VA–VA angle (°)	56.75 (42.23, 71.60)	60.80 (49.15, 75.05)	57.90 (41.15, 71.10)	0.383

VA, vertebral artery. Bold values denote statistical significance (*P* < 0.05). ^a^Group A was compared with Group B (*P* > 0.05). ^b^Group C was compared with Group A and Group B (*P* ≤ 0.05).

### Correlation between altitude and morphological characteristics of the vertebrobasilar artery

We further examined the association between the altitude and the morphological characteristics of the vertebrobasilar artery using the Spearman correlation analysis. Our results suggest that there is a relatively weak positive correlation between the altitude and the three characteristics of the vertebrobasilar artery, such as the tortuosity of the BA (*r*_s _= 0.190, *P *= 0.005), the lateral–mid–BA angle (*r*_s _= 0.201, *P *= 0.003), and the BA–VA angle (*r*_s _= 0.183, *P* = 0.006), all of which exhibit significant differences.

## Discussion

The plateau region is characterized by low oxygen, low air pressure, cold, high radiation, and significant day–night temperature differences. Long-term exposure to a hypoxic environment can affect gene expression, cellular metabolism, and physiological and biochemical functions in the human body (12). Hypoxic environments mainly affect hemodynamics by increasing the red blood cell count, hemoglobin, and blood viscosity and also lead to compensatory opening of the microcirculation (13, 14). Native highlanders have been reported to show earlier cardiovascular degeneration changes with ageing, particularly arterial wall stiffness (15). Bruno et al. (16) showed that the diameters of the carotid lumen were significantly larger in high-altitude residents than those in sea-level residents (6.98 ± 1.07 mm vs. 6.81 ± 0.85 mm, *P *= 0.004). In order to adapt to hypobaric hypoxia at high altitude, chronic vascular remodeling may occur (17). Therefore, our aim for this study is to examine the potential effect of chronic exposure to hypoxia on the vascular structure of the native highlanders who were born and permanently resided at high altitude.

High altitude is defined as an elevation over 2,500 masl (18). Current medical studies tend to group individuals based on fixed elevation ranges, and a limited research on a large range of altitude levels has been found. For this study, the altitude range of residents included individuals residing at elevations ranging from 1,800 masl in Minhe County to 4,403 masl in Gado County. Although our categorization of altitude (1,800–2,500 masl; 2,500–3,500 masl; and ≥3,500 masl) lacks a sufficient support, it is reasonable to observe the differences in vertebrobasilar morphology based on a gradient of approximately 1,000 masl.

In this study, the hypoxia environment at high altitude had an effect on the morphology of the posterior circulation artery. Among the many indexes of morphology, the lateral–mid–BA angle (23.18°_ _± 9.53° vs. 26.05°_ _± 10.10° vs. 31.07°_ _± 15.12°, *P *= 0.007) and the BA–VA angle (32.98° ± 17.85° vs. 34.51° ± 17.96° vs. 41.51° ± 19.22°, *P *= 0.024) significantly increased with increasing altitude, and the significant differences were observed mainly between group A and group C, indicating that the vertebrobasilar artery morphology significantly changed in individuals residing at altitudes over 3,500 masl. These findings are significantly larger than those reported by Jeong et al. (19) (the lateral–mid–BA angle is 21.4°, and the BA–VA angle is 14.00° on average). We also found a relatively weak positive correlation between the altitude and the tortuosity of the BA (*r*_s _= 0.190, *P *= 0.005), the lateral–mid–BA angle (*r*_s _= 0.201, *P *= 0.003), and the BA–VA angle (*r*_s _= 0.183, *P *= 0.006). The above-mentioned results suggest that an increase in altitude may lead to an increase in the sagittal angles of the vertebrobasilar artery, potentially complicating the hemodynamics of each vertebral artery into the basilar artery.

The increase in altitude was found to affect the tortuosity of the BA (1.05 ± 0.06 vs. 1.06 ± 0.08 vs. 1.10 ± 0.13, *P *= 0.005), and the results showed a significant difference between groups A and C (*P *= 0.010) and between groups B and C (*P *= 0.016). A study has demonstrated that the tortuosity and convexity of the BA are independently associated with the presence, burden, and distribution of plaques in the BA (20). In addition, the incidence of multiple bends in VA at an altitude of ≥3,500 masl was significantly higher than that in groups A and B. The number of bends in the bilateral VA intracranial segment was also associated with the presence of BA plaque (8). It has been demonstrated that vascular curvature is a geometrical feature that has a considerable influence on local blood flow patterns (21), which in turn affects the risk of atherosclerosis. This may be due to the fact that the areas of low wall shear stress and high oscillatory shear index were both larger in blood vessels with higher curvature. Both low and oscillatory wall shear stresses have negative effects on the endothelial function, phenotype, and wall permeability, thereby rendering the vascular wall more prone to the development of atherosclerosis (22). In this study, vascular morphology is more variable over 3,500 masl. The increase of tortuosity and curvature of the vertebrobasilar artery may affect the development of posterior circulation atherosclerosis in people at high altitude. Vascular morphology assessment in high-altitude population may be helpful in early predicting the presence of BA plaque and subsequent ischemic events in the posterior circulation territory.

Among the four basic geometric configurations of the vertebrobasilar artery, walking geometry accounted for 41.9% (93 patients), and the VA–VA angle of walking was the least (average 56.27° ± 18.83°). Tuning fork, lambda, and no confluence geometries accounted for 32.0%, 22.5%, and 3.6%, respectively. A study has shown that there are significant differences in the prevalence of BA plaque among the four configurations. Specifically, the prevalence of BA plaque was highest in the walking geometry and lowest in the tuning fork geometry (8). The walking geometry is characterized by the fact that two VAs bend in the same direction before merging into the basilar artery. Therefore, the inflow ratio and the vertebral artery curvature proximal to the confluence collectively influence the flow patterns and velocity profiles in the BA (7), and the blood flow from the two VAs impinges on the wall of the BA for a long time, causing the BA flow to curve in the opposite direction and making the hemodynamics complex (7). These chronic processes may induce BA curvature and plaque formation. In the tuning fork geometry, a primary feature is that the angles of approach between each vertebral and the basilar artery are roughly equal. As a result, the blood flows in the straight BA are roughly parallel, which reduces the probability of BA curvature and BA plaque formation (7). However, when the BA is curved, the BA curvature likely influences the basilar flow mixing and the shape of the axial velocity profiles (7). The lambda geometry is characterized by diameter differences of at least 0.3 mm between the two VAs. The asymmetric flow pattern formed by this geometry may be an important mechanical force for the formation of vertebrobasilar artery curvature, and it is also one of the pathogenic factors of vertebrobasilar artery junction infarction (23). The no confluence geometry may be caused by the obstruction of embryonic development (VA intracranial segment is directly extended to the posterior inferior cerebellar artery). This may lead to the intracranial distal segment of the vertebral artery. The incidence of no confluence geometry in this study is approximately 3.6%. In this geometry, the blood flow of a unilateral VA acts long term on the wall of the BA, which can also increase the curvature of the BA. Studies have shown that the asymmetry and resistance index of blood flow in no confluence geometry are higher, while the mean blood flow velocity is lower. This may be a negative hemodynamic factor of posterior circulation and may be related to stroke or transient ischemic attack (24).

Vertebral artery hypoplasia means that the unilateral vertebral artery is slender, and its diameter is significantly smaller than that of the contralateral side. In this study, a high detection rate of VAH was found at high altitude, with 32.0% (71 patients) of patients detected. VAH is more likely to occur in the right VA, accounting for approximately 57.7% and in the left vertebral artery for 42.3% of patients. The percentage of male and female detection was 63.4% (45 patients) and 36.6% (26 patients), respectively. VAH is not only an anatomical variant, but it may also affect the hemodynamics of the posterior circulation. It has been shown that the congenital asymmetric blood flow of the VA gradually bends and prolongs the BA in an asymmetric way, causing the BA to bulge to the opposite side of the dominant VA (19). This becomes the cause of posterior circulation ischemic events. VAH is considered to be an independent risk factor for PCI (25). Therefore, screening and follow-up observation of posterior circulation morphology will help avoid or reduce the occurrence of posterior circulation-related adverse events and contribute to the investigation of the etiology of vertigo in plateau areas.

It is worth mentioning that we used the GSI scanning protocol to study morphological differences in the vertebrobasilar artery. Energy-spectrum CT introduces the concept of multiparameter imaging by using a single-source instantaneous kV switching technology (140 and 80 kV). This technology samples two energy data simultaneously, and multiple single-energy images within this energy range are obtained. Studies have shown that the signal-to-noise ratio of blood vessels and surrounding tissues can be improved at approximately 60 keV (26). In this study, we chose a single-energy level of 40–60 keV, and the energy value was adjusted autonomously within the range of the optimal head–neck CTA to obtain high-quality images of the vertebrobasilar artery accurately. Energy-spectrum CT, using ASiR-V 50% combined with virtual single-energy imaging technology, can not only effectively reduce the radiation dose but also improve the image quality and the vascular CT value and contrast and meet the needs of subsequent image postprocessing and morphological evaluation.

From a perspective of morphological variation, the increase in VAS curvature may exhibit negative effects on the occurrence and development of posterior circulation diseases. However, from a macro perspective, hypoxia is not necessarily deleterious. In fact, longevity at high altitude has been proven (27), and small vessel compensation and physiological changes in chronic hypoxic environments are often beneficial. Nitric oxide is one of the primary molecules responsible for vasodilatation (28). It has been reported that hypoxia increases the production of serum nitric oxide (29). Hypoxia at high altitude promotes vascular endothelial growth factor (VEGF) deposition, which may contribute to choroidal neovascularization and angiogenesis in other tissues (30). Study has shown that greater vascularity of the retina in people at high altitude (31). Exposure to high altitude can even improve myocardial perfusion in patients with coronary heart disease (32). When intravascular hemodynamics change for a long time, arterial morphological or structural adaptation can occur to minimize the effect of changed hemodynamics on the vascular wall, including changes in diameter and wall thickness (33, 34). These studies suggest that hypoxia promotes the opening of collateral circulation and positive vascular remodeling of systemic arteries for adaptive compensation. Therefore, the influence of the adaptive changes in VAS anatomy on the posterior circulation diseases in high-altitude residents needs further exploration.

Some limitations in our study need consideration. First, this study was a cross-sectional study in a single center, our data were limited, and a selection bias might be unavoidable. Second, there was no direct study of intravascular hemodynamics in our work, and the underlying mechanism of altitude that influences the morphological structure of VAS needs to be investigated from the hemodynamic aspect in future studies.

## Conclusion

The above-mentioned results demonstrate that the higher the altitude, the greater the tortuosity of BA and vertebrobasilar artery sagittal angle, as well as the number of VA multibending groups. Long-term exposure to a plateau hypoxic environment can induce alterations in the morphology of the vertebrobasilar artery. These changes may be associated with the adaptive response of long-term residents to the low-pressure and low-oxygen environment, such as the increase in the red blood cell count and changes in the rate of the blood flow. They may also further modify the hemodynamics in the vasculature. The morphological changes increase the impact of blood flow on the blood vessel wall of the tortuous segment and further affect its structural shape. This characteristic change is of significant relevance to provide an anatomical basis for clarifying the pathogenesis of vertigo and posterior circulation ischemic disease in people living at high altitude.

## Data Availability

The original contributions presented in the study are included in the article/supplementary material, further inquiries can be directed to the corresponding author.

## References

[1] Yang Y Liang C Shen C Tang H Ma S Zhang Q The effects of pharmaceutical thrombolysis and multi-modal therapy on patients with acute posterior circulation ischemic stroke: results of a one center retrospective study. Int J Surg. (2017) 39:197–201. 10.1016/j.ijsu.2017.02.002 28185942

[2] Connell L Koerte IK Laubender RP Morhard D Linn J Becker HC Hyperdense basilar artery sign: a reliable sign of basilar artery occlusion. Neuroradiology. (2012) 54(4):321–7. 10.1007/s00234-011-0887-6 21584673

[3] Caplan L Chung CS Wityk R Glass T Tapia J Pazdera L New England medical center posterior circulation stroke registry: I. Methods, data base, distribution of brain lesions, stroke mechanisms, and outcomes. J Clin Neurol. (2005) 1(1):14–30. 10.3988/jcn.2005.1.1.14 20396469 PMC2854928

[4] Yu J Zhang S Li ML Ma Y Dong YR Lou M Relationship between the geometry patterns of vertebrobasilar artery and atherosclerosis. BMC Neurol. (2018) 18(1):83. 10.1186/s12883-018-1084-6 29895279 PMC5996488

[5] Deng D Cheng FB Zhang Y Zhou HW Feng Y Feng JC. Morphological analysis of the vertebral and basilar arteries in the Chinese population provides greater diagnostic accuracy of vertebrobasilar dolichoectasia and reveals gender differences. Surg Radiol Anat. (2012) 34(7):645–50. 10.1007/s00276-012-0960-9 22427028

[6] Ma G Yu Y Duan H Dou Y Jia Y Zhang X Subtraction CT angiography in head and neck with low radiation and contrast dose dual-energy spectral CT using rapid kV-switching technique. Br J Radiol. (2018) 91(1086):20170631. 10.1259/bjr.20170631 29412008 PMC6223275

[7] Wake-Buck AK Gatenby JC Gore JC. Hemodynamic characteristics of the vertebrobasilar system analyzed using MRI-based models. PLoS One. (2012) 7(12):e51346. 10.1371/journal.pone.0051346 23251503 PMC3519605

[8] Zheng J Sun B Lin R Teng Y Zhao X Xue Y. Association between the vertebrobasilar artery geometry and basilar artery plaques determined by high-resolution magnetic resonance imaging. BMC Neurosci. (2021) 22(1):20. 10.1186/s12868-021-00624-5 33765922 PMC7992992

[9] Thierfelder KM Baumann AB Sommer WH Armbruster M Opherk C Janssen H Vertebral artery hypoplasia: frequency and effect on cerebellar blood flow characteristics. Stroke. (2014) 45(5):1363–8. 10.1161/strokeaha.113.004188 24699051

[10] Zhang DP Peng YF Zhang HL Ma JG Zhao M Yin S Basilar artery tortuosity is associated with white matter hyperintensities by TIMP-1. Front Neurosci. (2019) 13:836. 10.3389/fnins.2019.00836 31474817 PMC6703195

[11] Kim BJ Kim HY Jho W Kim YS Koh SH Heo SH Asymptomatic basilar artery plaque distribution and vascular geometry. J Atheroscler Thromb. (2019) 26(11):1007–14. 10.5551/jat.47365 30918163 PMC6845693

[12] Alam P Agarwal G Kumar R Mishra A Saini N Mohammad G Susceptibility to high-altitude pulmonary edema is associated with circulating miRNA levels under hypobaric hypoxia conditions. Am J Physiol Lung Cell Mol Physiol. (2020) 319(2):L360–8. 10.1152/ajplung.00168.2020 32692577

[13] Tanaka M Nakamura S Maekawa M Higashiyama S Hara H. ANKFY1 is essential for retinal endothelial cell proliferation and migration via VEGFR2/Akt/eNOS pathway. Biochem Biophys Res Commun. (2020) 533(4):1406–12. 10.1016/j.bbrc.2020.10.032 33092793

[14] Nakamura-Utsunomiya A Tsumura M Okada S Kawaguchi H Kobayashi M. Downregulation of endothelial nitric oxide synthase (eNOS) and endothelin-1 (ET-1) in a co-culture system with human stimulated X-linked CGD neutrophils. PLoS One. (2020) 15(4):e0230665. 10.1371/journal.pone.0230665 32251485 PMC7135077

[15] Otsuka K Norboo T Otsuka Y Higuchi H Hayajiri M Narushima C Chronoecological health watch of arterial stiffness and neuro-cardio-pulmonary function in elderly community at high altitude (3,524 m), compared with Japanese town. Biomed Pharmacother. (2005) 59(Suppl 1):S58–67. 10.1016/s0753-3322(05)80012-5 16275510 PMC2819461

[16] Bruno RM Cogo A Ghiadoni L Duo E Pomidori L Sharma R Cardiovascular function in healthy Himalayan high-altitude dwellers. Atherosclerosis. (2014) 236(1):47–53. 10.1016/j.atherosclerosis.2014.06.017 25014034

[17] Lewis NC Bailey DM Dumanoir GR Messinger L Lucas SJ Cotter JD Conduit artery structure and function in lowlanders and native highlanders: relationships with oxidative stress and role of sympathoexcitation. J Physiol. (2014) 592(5):1009–24. 10.1113/jphysiol.2013.268615 24324004 PMC3948560

[18] Gaur P Sartmyrzaeva M Maripov A Muratali Uulu K Saini S Ray K Cardiac acclimatization at high altitude in two different ethnicity groups. High Alt Med Biol. (2021) 22(1):58–69. 10.1089/ham.2020.0035 33400909

[19] Jeong SK Lee JH Nam DH Kim JT Ha YS Oh SY Basilar artery angulation in association with aging and pontine lacunar infarction: a multicenter observational study. J Atheroscler Thromb. (2015) 22(5):509–17. 10.5551/jat.26245 25421902

[20] Deng S Zheng J Wu Y Yang D Chen H Sun B Geometrical characteristics associated with atherosclerotic disease in the basilar artery: a magnetic resonance vessel wall imaging study. Quant Imaging Med Surg. (2021) 11(6):2711–20. 10.21037/qims-20-1291 34079735 PMC8107302

[21] Lee KE Lee JS Yoo JY. A numerical study on steady flow in helically sinuous vascular prostheses. Med Eng Phys. (2011) 33(1):38–46. 10.1016/j.medengphy.2010.09.005 20933454

[22] Li X Liu X Li X Xu L Chen X Liang F. Tortuosity of the superficial femoral artery and its influence on blood flow patterns and risk of atherosclerosis. Biomech Model Mechanobiol. (2019) 18(4):883–96. 10.1007/s10237-019-01118-4 30652210

[23] Hong JM Chung CS Bang OY Yong SW Joo IS Huh K. Vertebral artery dominance contributes to basilar artery curvature and peri-vertebrobasilar junctional infarcts. J Neurol Neurosurg Psychiatry. (2009) 80(10):1087–92. 10.1136/jnnp.2008.169805 19414436 PMC2735647

[24] Liu IW Ho BL Chen CF Han K Lin CJ Sheng WY Vertebral artery terminating in posterior inferior cerebellar artery: a normal variation with clinical significance. PLoS One. (2017) 12(4):e0175264. 10.1371/journal.pone.0175264 28394897 PMC5386266

[25] Hu XY Li ZX Liu HQ Zhang M Wei ML Fang S Relationship between vertebral artery hypoplasia and posterior circulation stroke in Chinese patients. Neuroradiology. (2013) 55(3):291–5. 10.1007/s00234-012-1112-y 23117257

[26] Schneider D Apfaltrer P Sudarski S Nance JW Haubenreisser H Fink C Optimization of kiloelectron volt settings in cerebral and cervical dual-energy CT angiography determined with virtual monoenergetic imaging. Acad Radiol. (2014) 21(4):431–6. 10.1016/j.acra.2013.12.006 24594412

[27] Zubieta-Calleja GR Zubieta-Deurioste NA. Extended longevity at high altitude: benefits of exposure to chronic hypoxia. BLDE Univ J Health Sci. (2017) 2(2):80. 10.4103/bjhs.bjhs_7_17

[28] Christopherson KS Bredt DS. Nitric oxide in excitable tissues: physiological roles and disease. J Clin Invest. (1997) 100(10):2424–9. 10.1172/jci119783 9366555 PMC508441

[29] Xu XP Pollock JS Tanner MA Myers PR. Hypoxia activates nitric oxide synthase and stimulates nitric oxide production in porcine coronary resistance arteriolar endothelial cells. Cardiovasc Res. (1995) 30(6):841–7. 10.1016/S0008-6363(95)00117-4 8746197

[30] Klettner A Kampers M Töbelmann D Roider J Dittmar M. The influence of melatonin and light on VEGF secretion in primary RPE cells. Biomolecules. (2021) 11(1):114. 10.3390/biom11010114 33467052 PMC7830335

[31] Acosta C Gloria JM Lavaque A García V Torres E Agüero C Relationship of geographic altitude with foveal avascular zone metrics and vascular density values assessed by OCT angiography. Ophthalmol Retina. (2020) 4(4):394–402. 10.1016/j.oret.2019.10.017 31956074

[32] del Pilar Valle M García-Godos F Woolcott OO Marticorena JM Rodríguez V Gutiérrez I Improvement of myocardial perfusion in coronary patients after intermittent hypobaric hypoxia. J Nucl Cardiol. (2006) 13(1):69–74. 10.1016/j.nuclcard.2005.11.008 16464719

[33] Langille BL O'Donnell F. Reductions in arterial diameter produced by chronic decreases in blood flow are endothelium-dependent. Science. (1986) 231(4736):405–7. 10.1126/science.3941904 3941904

[34] Korshunov VA Berk BC. Flow-induced vascular remodeling in the mouse: a model for carotid intima-media thickening. Arterioscler Thromb Vasc Biol. (2003) 23(12):2185–91. 10.1161/01.Atv.0000103120.06092.14 14576075

